# 

**Published:** 1912-10

**Authors:** 


### CONTENTS.

ORIGINAL COMMUNICATIONS:—

A Report of Wednesday's Surgical Clinic in the Atlanta School of Medicine. By Edward G. Jones, A.B., M.D., and Dean F. Winn, M.D., Atlanta, Ga ..... 345

Etiologic Factors and Recurrent Attacks of Pellagra By Geo. C. Mizell, M.D., Atlanta, Ga ..... 366

Hernias of the Utterine Appendages. By Aime Paul Heineck, M.D ..... 380

EDITORIAL ..... 396

SELECTIONS AND ABSTRACTS ..... 397

BOOK REVIEWS ..... 405

